# Targeting the Lnc-OPHN1-5/androgen receptor/hnRNPA1 complex increases Enzalutamide sensitivity to better suppress prostate cancer progression

**DOI:** 10.1038/s41419-021-03966-4

**Published:** 2021-09-20

**Authors:** Meng Zhang, Yin Sun, Chi-Ping Huang, Jie Luo, Li Zhang, Jialin Meng, Chaozhao Liang, Chawnshang Chang

**Affiliations:** 1 Department of Urology, The First Affiliated Hospital of Anhui Medical University, Institute of Urology, & Anhui Province Key Laboratory of Genitourinary Diseases, Anhui Medical University, Hefei, China; 2 George Whipple Lab for Cancer Research, Departments of Pathology, Urology, Radiation Oncology, The Wilmot Cancer Institute, University of Rochester Medical Center, Rochester, NY USA; 3 Institute of Urology, Shenzhen University, Shenzhen, China; 4 Department of Urology, China Medical University, Taichung, Taiwan

**Keywords:** Prostate cancer, Drug regulation

## Abstract

Long non-coding RNAs (lncRNAs) have been found to play critical roles in regulating gene expression, but their function in translational control is poorly understood. We found lnc-OPHN1-5, which lies close to the androgen receptor (AR) gene on chromosome X, increased prostate cancer (PCa) Enzalutamide (Enz) sensitivity via decreasing AR protein expression and associated activity. Mechanism dissection revealed that lnc-OPHN1-5 interacted with AR-mRNA to minimize its interaction with the RNA binding protein (RBP) hnRNPA1. Suppressing lnc-OPHN1-5 expression promoted the interaction between AR-mRNA and hnRNPA1, followed by an increase of ribosome association with AR-mRNA and translation. This effect was reversed by increasing lnc-OPHN1-5 expression. Consistently, the in vivo mice model confirmed that knocking down lnc-OPHN1-5 expression in tumors significantly increased the tumor formation rate and AR protein expression compared with the control group. Furthermore, knocking down hnRNPA1 blocked/reversed shlnc-OPHN1-5-increased AR protein expression and re-sensitized cells to Enz treatment efficacy. Evidence from Enz-resistant cell lines, patient-derived xenograft (PDX) models, clinical samples, and a human PCa study accordantly suggested that patients with low expression of lnc-OPHN1-5 likely have unfavorable prognoses and probably are less sensitive to Enz treatment. In summary, targeting this newly identified lnc-OPHN1-5/AR/hnRNPA1 complex may help develop novel therapies to increase Enz treatment sensitivity for suppressing the PCa at an advanced stage.

## Introduction

Prostate cancer (PCa) is the most frequently diagnosed malignancy among men in the western world [1–4]. The androgen-deprivation therapy (ADT) with recently developed anti-androgen Enzalutamide (Enz) is frequently followed by the development of ADT-resistance. An important molecular mechanism of ADT-resistance is the continued activation of the androgen receptor (AR) protein. Identifying novel biological mechanisms underlying this aberrant AR activation holds great promise to improve the treatment of castration-resistant prostate cancer (CRPC) patients.

Previous studies have shown mutations, genomic amplifications, and deletions, which were commonly found in the AR gene during the development of CRPC, ultimately contributed to the continued and enhanced AR activity [5, 6]. More recent studies indicated that enhancers located much farther away on the linear sequence might activate AR transcription through chromatin looping to drive the progression of metastatic CRPC [7]. Simultaneously, the altered chromatin landscape around the AR locus may also change the expression of resident genes, including long non-coding RNAs (lncRNAs).

The lncRNAs are RNA transcripts of more than 200 nt long but have little ability to encode proteins. More recently, numerous lncRNAs have been annotated, and their functions in regulating biological procedures have begun to be uncovered [8–10]. The lncRNAs could be associated with chromatin-modifying proteins, such as the polycomb repressive complexes (PRC2), to regulate chromatin states [11, 12]. Accumulating evidence found that lncRNAs are able to regulate cellular processes *via* post-translational mechanisms [13, 14], in addition to their ability in regulating the mRNA translation by interacting with RNA binding proteins (RBPs), such as hnRNPA1, thus influencing the level of the encoded protein and biological functions of downstream genes [15, 16]. Based on these regulatory mechanisms, lncRNAs have been shown to play pivotal roles in human cancer development.

Here, we report a novel lncRNA, lnc-OPHN1-5, which was located one megabase (MB) 3’ of AR on chromosome X, and it influenced Enz treatment sensitivity *via* decreasing AR mRNA translation, thus decreased AR protein level and transcriptional activity. Targeting this newly identified lnc-OPHN1-5/AR/hnRNPA1 complex with silenced hnRNPA1 (shhnRNPA1) led to increasing Enz treatment sensitivity for greater suppression of CRPC progression.

## Materials and methods

### Human samples

We collected the formalin-fixed, paraffin-embedded (FFPE) samples from 75 prostate cancer patients with complete follow-up features from the Department of Urology, The First Affiliated Hospital of Anhui Medical University. The biochemical recurrence (or recurrence-free survival) was defined as the prostate-specific antigen (PSA) level higher than 0.2 ng/mL determined 2–4 months after radical prostatectomy, and confirmation tests were performed to prove the continued elevation of PSA levels. The ethics of our study were approved by The Committee on Medical Ethics of The First Affiliated Hospital of Anhui Medical University (J2019911 & PJ 20210214). The tissues were used to extract RNA (Cat. 73504, RNeasy FFPE Kit, Hilden, Germany) for lnc-OPHN1-5 quantification.

### Cell lines, inhibitors, and antibodies

The information of cell lines used in current work was demonstrated in our previous work [17], including C4-2R (derived from C4-2 cells and resistant to Enz treatment) and the C4-2BR (derived from C4-2B cells and resistant to Enz treatment) cells, which were cultured in RPMI media supplied with 10 and 20 µM Enz, respectively. Antibodies of GAPDH (sc-47724), α-Tubulin (sc-134241), AR (N-20), hnRNPA1 (sc-32301), hnRNPA/B (sc-376411), hnRNPK (sc-28380), and HUR (sc-5261) were purchased from Santa Cruz Biotechnology (Santa Cruz Biotechnology, TX, USA). We also purchased an AR antibody (3202S) from Cell Signaling Technology (Cell Signaling Technology, MA, USA). The lnc-OPHN1-5 and AR-specific biotins were purchased from Integrated DNA Technologies Company (Integrated DNA Technologies, CA, USA).

### Plasmids and lentivirus packaging

The plasmids used in the current work were listed in Table S1, and they were packaged with the psAX2 packaging plasmid and pMD2G envelope plasmid and co-transfected into 293T cells via the standard calcium chloride transfection method. After 48 h, we collected the lentivirus supernatants and kept them in −80 C° freezer for future use. For the viruses that were generated by pLKO.1 vector-based plasmids, cells were selected for stable expression with puromycin (1.5 µg/mL) (CAS № 58-58-2, Cayman, MI, USA).

### Western blotting (WB) and mouse mammary tumor virus (MMTV)-luciferase assay

Briefly, we used cell lysis buffer to lyse collected cells, and after quantification and loading-mixed boiling processes, equal weights of proteins were separated on 10% sodium dodecyl sulfate/polyacrylamide gel electrophoresis gel and then transferred onto PVDF membranes (Millipore, MA, USA). We used 5% milk to block the membrane for 1 h and then incubated it with the specific primary antibodies overnight. After washing, the blots were incubated with HRP-conjugated secondary antibodies and then visualized using the ECL system (Thermo Fisher Scientific, Rochester, NY, USA). The AR activity was determined by MMTV-luciferase assay according to our previous work [18, 19].

### MTT assay and ethynyl-2-deoxyuridine (EdU) staining

Cells were mixed with RPMI media, and we added 500 µl media mix containing 5000 cells/well into the 24-well plates. Before collecting, we added 50 µL MTT reagent (5 mg/mL MTT in PBS, Amresco, OH, USA) to each well and incubated at 37 °C for 2 h. After removing supernatants, we added 1 mL dimethyl sulfoxide (Amresco, OH, USA) to dissolve the crystals. The optical density (OD) was determined at a wavelength of 570 nm on a microplate reader. The effects of lnc-OPHN1-5 on PCa cell Enz sensitivity were also tested by EdU incorporation assay using Cell-Light™ EdU Apollo^®^567 In Vitro Imaging Kit (RiboBio, Guangzhou, China). Briefly, the EdU was added to each well with a final concentration of 50 μM. After 2 h incubation, cells were fixed with 4% paraformaldehyde at room temperature (RT). We used PBST [phosphate buffer saline (PBS) with 0.1% Triton X-100] to wash each well 3 times, followed by 1 × Apollo solution incubating for 30 min at RT away from light. Finally, the cell nuclei were stained with 1 × Hoechst for 30 min, and the stained cells were visualized by fluorescence microscopy (Olympus, Tokyo, Japan).

### Ribosome-enriched RNA extraction and nucleus-cytoplasm fraction

C4-2 cells (2 × 107) were collected, and centrifuged for 5 min at 900 × *g*, and washed twice with pre-chilled 0.15 M NaCl. The detailed steps followed T Masse et al.’s [20] study. We resuspended these cells in 2 mL of buffer (0.25 M KCl, 0.005 M MgCI_2_, 0.014 M 2-mercaptoethanol (2-ME), 0.05 M Tris-HCl/pH 7.4, 0.25 M sucrose) and lysed by adding 70 μL of 20% Nonidet P-40 solution [21]. After 15 min at 4 °C, we removed the nuclei by centrifuging for 10 min at 900 × *g*. Then we removed the mitochondria by high-speed centrifugation at 12,500 × *g* for 10 min. We adjusted the supernatant to 0.5 M KCl and layered on to 2 mL cushion (1 M sucrose made in 0.5 M KCl, 0.005 M MgCl_2_, 0.05 M Tris-HCl/pH 7.4). Ribosomes were pelleted by centrifugation for 4 h at 4 °C and 260,000 × *g*. We re-suspended the pellets with 500 μL Trizol (Invitrogen, CA, USA) for the next step of the RNA extraction process.

Cytoplasmic and nuclear RNAs were purified from C4-2 cells using a cytoplasmic and nuclear RNA purification kit (Norgen Biotek, ON, Canada). Briefly, 400 μL lysis buffer was added to the C4-2 cell pellet and incubated on ice for 10 min, and then centrifuged for 15 min at 14,000 rpm to separate the cellular fractions. The supernatant comprised a cytoplasmic fraction, while the pellet comprised the nuclear component. We mixed these two tubes with 800 μL 1.6 M sucrose solution and carefully layered them on the surface of 2 separate 1000 μL sucrose solution tubes. We centrifuged the two fractions at 14,000 rpm for 15 min (4 °C), and then the cytoplasm was obtained from the top layer of the sucrose cushion. We purified the cytoplasm RNA according to the protocol of the Norgen kit. We obtained the nuclear pellet from the bottom of the tube and used 400 μL 1× PBS to wash it. The nuclear pellet was collected after another centrifugation process at 14,000 rpm for 5 min, and then the nuclear RNA was purified according to the protocol of the Norgen kit. The purified RNA was re-suspended in 500 μL Trizol (Invitrogen).

### RNA immunoprecipitation

We added 1 mL RNase-free cell lysis buffer to the plates of cells, along with 1 µL RNase inhibitor (cat# M0307S, NEB, MA, USA). After storage in −80 °C freezer for 30 min, the cell lysates were centrifuged at 14,000 rpm for 15 min to collect the supernatants. Then, the cell lysates were mixed with Streptavidin-coupled Dynabeads (Invitrogen) and incubated at 4 °C for 2 h with gentle rotation. Each of the lysates was divided into three parts, including input, control, and test groups. The antibodies or biotins were added to each element as needed. After 12 h incubation, we added 20 μL Streptavidin-coupled Dynabeads to each tube and rotated for 1 h. These beads were washed by RIP buffer 10 times and re-suspended in 500 μL Trizol for RNA extraction. The RNA extraction and reverse transcription-polymerase chain reaction (RT-PCR) protocol were performed according to our previous work [22].

### Animal model and patient-derived xenografts

The ethics of animal studies were approved by the Ethics Committee of Anhui Medical University. The 6-week-old male NOD CRISPR Prkdc Il2r Gamma triple-immunodeficient mice (NCG) were purchased from GemPharmatech Co., Ltd (Nanjing, China) [23]. The C4-2 cells, which expressed pLKO.1 or shlnc-OPHN1-5, were obtained by stable clone selection procedures. Cells (3 ×106) mixed with Matrigel (1:1, v/v, Cat. 356234, BD, USA) were subcutaneously injected into the NCG male mice at 6 weeks of age (randomly assigned into two groups). The mouse body weights and tumor volumes were monitored weekly, and the tumor weights were determined after sacrifice [24]. In addition, the patient-derived xenograft tissues (PDX) were gifts from the University of MD Anderson (USA) and subcutaneously injected into the NOD-SCID male mice as above. After a long-term treatment with Enz or vehicle, the tumor tissues were obtained for RNA extraction, immunohistochemical (IHC), and RT-PCR process.

### IHC staining

We obtained the tumor tissues from each mouse and embedded them in paraffin. These samples were cut into 5 µm thick slices and dehydrated with xylene. We stained the AR (#8428, Cell Signaling Technology) and hnRNPA1 (Abcam, Cambridge, UK) antibodies at 4 °C incubator overnight and then incubated with the secondary antibody at RT for 1 h. The detailed steps of the IHC process and the score quantification were described in our previous work [25, 26]. The IHC scores were determined by *P* x *I* [positively stained cells (*P*%) x the intensity (*I*), range from 1 = weak; 2 = moderate to 3 = strong. IHC score ≤ 300.]

### Statistical analysis

The results for each treatment group were presented as a representative of multiple repeated experiments with each data point performed in triplicate. Statistical studies between the two groups were conducted using the two-tailed unpaired Student’s *t*-test or non-parametric test (Mann–Whitney test) depending on the homogeneity test of variance. Data were presented as the mean and standard error of the mean (mean ± SEM) or the mean+/-standard deviation (mean+/-SD) unless mentioned otherwise. A *P*-value < 0.05 was considered statistically significant.

## Results

### Identifying lnc-OPHN1-5 as a regulator of Enz treatment sensitivity in CRPC

Previous studies reported that aberrations of AR (including mutation, deletion, and amplification) are frequently found in PCa patients (Fig. S1) [27–29]. More recently, chromatin elements outside the AR locus have also been found to be able to contribute to the PCa progression via a long-distance transcriptional regulation of the AR gene [5]. These alterations likely will also influence the expression of genes, such as lncRNAs in close proximity of the AR locus, and whether their expression also contributes to the PCa progression, particularly resistance to the anti-androgen Enz is not known.

**Fig. 1:**
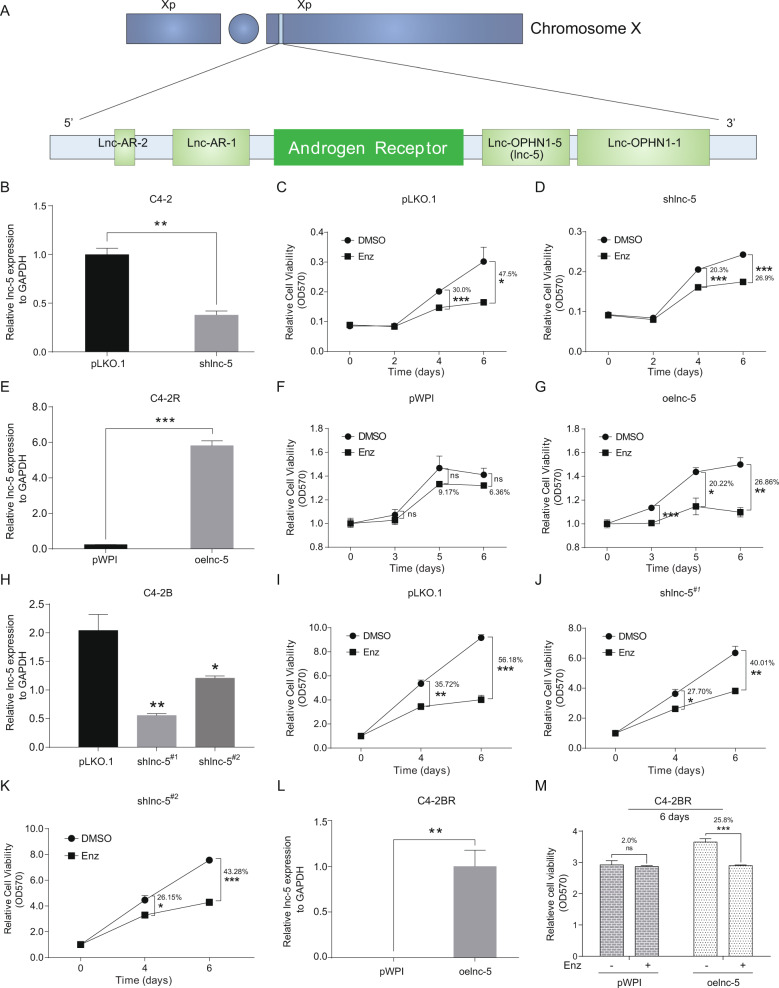
The lnc-OPHN1-5 (lnc-5) influences prostate cancer (PCa) cell Enzalutamide (Enz) treatment sensitivity. **A** The model showed the location of lncRNAs surrounding AR on Chromosome X. **B** The knocking down efficiency of lnc-5^#2^ (shlnc-5^#2^) in C4-2 cells. C-D. Compared to pLKO.1 (**C**), shlnc-5 in C4-2 cells expression significantly decreased Enz treatment sensitivity (**D**) indicated by MTT assay. **E**. Ectopic lnc-5 (oelnc-5) expression efficiency in C4-2R cells. **F**-**G** Compared to pWPI (**F**), oelnc-5 expression in C4-2R cells significantly increased Enz treatment sensitivity. **G** indicated by MTT assay. **H** Knock down efficiency of lnc-5 (shlnc-5^#1^ and shlnc-5^#2^) in C4-2B cells. **I**–**K** Compared to pLKO.1 (**I**), knocking down lnc-5, using shlnc-5^#1^ in (**J**) or shlnc-5^#2^ in (**K**) in C4-2B cells expression significantly decreased Enz treatment sensitivity indicated by MTT assay. **L** The oelnc-5 expression efficiency in C4-2BR cells. **M** The oelnc-5 expression significantly increased Enz treatment sensitivity in C4-2BR cells indicated by MTT assay. *P < 0.05; **P < 0.01; ***P < 0.001; ns, no significant difference.

To examine their potential role in regulating AR activity, we found four lncRNAs, lnc-AR-1, lnc-AR-2, lnc-OPHN1-1, and lnc-OPHN1-5, which were 1 MB 5’ and 3’ of AR on the X chromosome (Fig. 1A) based on the LNCipedia database (https://lncipedia.org/). To implicate their potential role in PCa progression, we generated four short hairpin RNAs (shRNAs) to knock down their expression to test whether there is a lncRNA that could influence Enz treatment sensitivity. The results revealed one lncRNA, lnc-OPHN1-5, whose silencing (sh), with two different shRNAs to eliminate off-target effects, significantly decreased Enz treatment sensitivity in C4-2 cells (Fig. 1B–D; Figs. S2A and S3A-B). In contrast, overexpression (oe) of lnc-OPHN1-5 significantly increased Enz treatment sensitivity in C4-2R cells (Fig. 1E–G). Consistent results were generated when we replaced the C4-2/C4-2R cells with C4-2B/C4-2BR cells (Fig. 1H–M). In addition, we used the EdU staining assay to confirm the MTT data, and similar regulation of PCa Enz treatment sensitivity by lnc-OPHN1-5 was observed (Fig. S2B). The analysis of the potential protein-coding capacity indicated that lnc-OPHN1-5 could not encode a protein (Fig. S3C). Since knowing the subcellular localization of lncRNAs will provide fundamental insights into their biological function and foster hypotheses for underlying mechanisms, we employed nuclear and cytoplasm fractionation assays and demonstrated that lnc-OPHN1-5 is located in both the nucleus and cytoplasm (Fig. S3D).

**Fig. 2:**
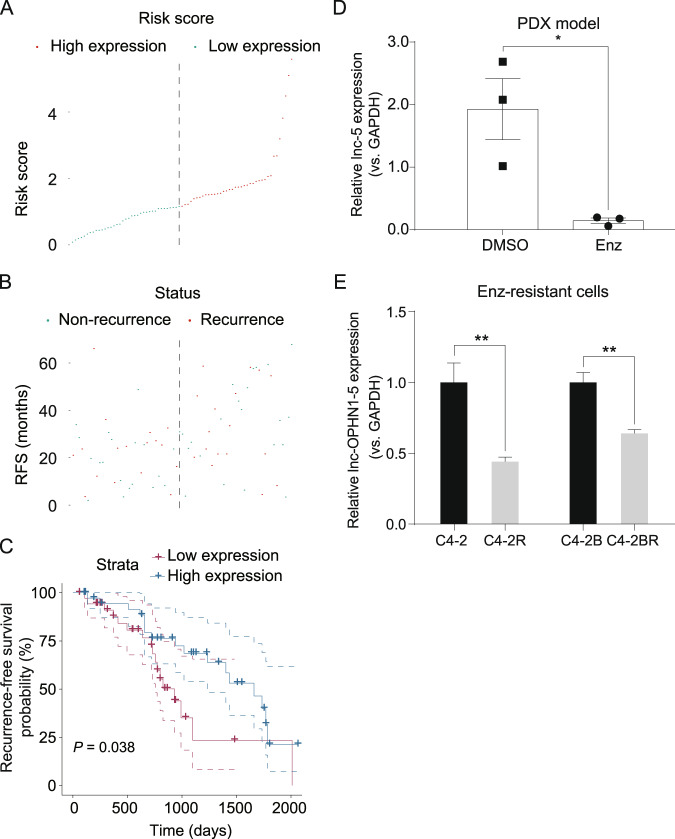
Clinical values of lnc-OPHN1-5 (lnc-5) in prostate cancer (PCa). **A** Sorting the PCa samples by lnc-5 expression determined by reverse transcription-polymerase chain reaction (RT-PCR) assay. **B** Displaying the recurrent status of PCa patients in high- and low-lnc-5 expression subgroups. **C** Kaplan–Meier plot and log-rank analysis suggested that the patients with lower expression of lnc-5 had worse recurrence-free survival outcomes than those with higher lnc-5 expression. **D** The relative lnc-5 expression in patient-derived Xenograft (PDX) models after long-time Enzalutamide (Enz)-treatment compared with controls determined by RT-PCR assay. **E** The relative lnc-5 expression in C4-2R & C4-2BR cells compared with parental C4-2 & C4-2B cells determined by RT-PCR assay. *P < 0.05; **P < 0.01.

To further determine the clinical significance of lnc-OPHN1-5, we tested its expression in clinical samples from PCa patients, Enz-resistant cell lines, and PDX models. Consistent with its biological function, we found that lower expression of lnc-OPHN1-5 was significantly associated with unfavorable prognosis of PCa patients (Table S2 and Fig. 2A–C). As indicated in the PDX mouse model of human PCa samples, we found that after long-term Enz treatment, the expression of lnc-OPHN1-5 was significantly decreased (Fig. 2D), which was consistent with the finding in C4-2/C4-2R & C4-2B/C4-2BR cells with long-term Enz treatment compared with their parental cells (Fig. 2E). We further applied the human clinical sample survey *via* Gene Expression Omnibus (GEO) dataset (GSE48403) analysis. Results revealed that in some PCa patients with a higher grade or higher Gleason score of PCa, their lnc-OPHN1-5 expressions were decreased after ADT (three of seven patients decreased, Fig. S3E and Table S3), and in contrast, in some PCa patients with a lower grade or lower Gleason score of PCa, their lnc-OPHN1-5 expressions were increased after ADT (four of seven patients increased, Fig. S3E), indicating that ADT efficacy might be linked with lnc-OPHN1-5 expression.

Taken together from Figs. 1–2, Table S2-S3, and Fig. S1-S3, these data suggest that the patients with low expression of lnc-OPHN1-5 likely have unfavorable prognoses and are probably less sensitive to Enz treatment.

### The lnc-OPHN1-5 can alter AR protein expression and transcriptional activity

**Fig. 3:**
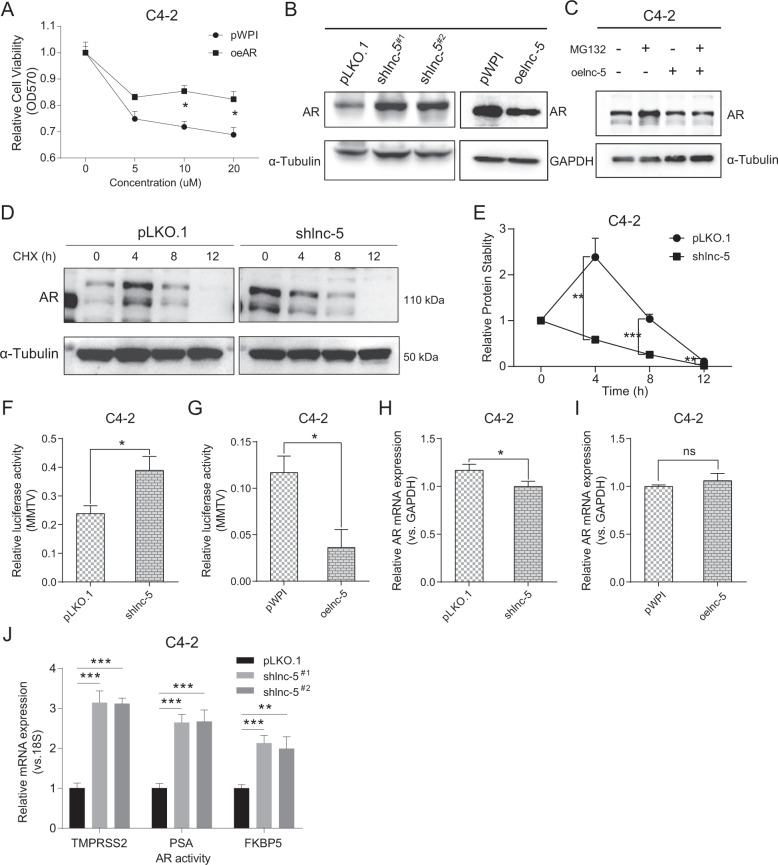
The lnc-OPHN1-5 (lnc-5) influences AR protein expression and transcriptional activity independent of influencing its protein stability. **A** Ectopic AR (oeAR) expression decreased Enzalutamide (Enz) treatment sensitivity in C4-2 cells determined by MTT assay. **B** After manipulating lnc-5 expression in C4-2 cells, the influence of AR protein expression was determined by western blotting (WB) assay. **C** The influence of proteasome inhibitor MG132 on AR protein expression before and after ectopic lnc-5 (oelnc-5) expression determined by WB assay. **D** The stability variation of AR protein with or without cycloheximide (CHX) treatment in C4-2 cells with knock down of lnc-5 (shlnc-5) expression compared to pLKO.1 as determined by WB assay. **E** The quantification results of AR protein degradation after shlnc-5 expression. **F**, **G** Shlnc-5 expression increased AR transcriptional activity tested by MMTV-luciferase assay, which could be suppressed by oelnc-5 expression in C4-2 cells. **H**, **I** Reverse transcription-polymerase chain reaction (RT-PCR) assay showed that shlnc-5 expression slightly decreased AR mRNA expression, while oelnc-5 expression did not influence the AR mRNA expression, indicating that lnc-5 regulated AR protein expression independent of AR mRNA expression. **J** Shlnc-5 expression significantly increased the AR classic downstream PSA, TMPRSS2, and FKBP5 expression determined by RT-PCR assay. *P < 0.05; **P < 0.01; ***P < 0.001; ns, no significant difference.

Several studies suggested that lncRNAs participate in the regulation of epigenetic, transcription, post-transcription, translation, and post-translational modification processes [30–32]. Since AR and AR signals have a significant impact on Enz treatment sensitivity [33, 34] and the lnc-OPHN1-5 is physically close to the Chromosome X on the *AR* gene, we were interested in determining whether lnc-OPHN1-5 might function *via* altering AR expression or activity to regulate Enz sensitivity. Consistent with previous findings that AR expression can directly impact Enz sensitivity (Fig. 3A), we found that knocking down lnc-OPHN1-5 increased AR protein expression (Fig. 3B), but failed to increase the protein stability (Fig. 3C–E). Ectopic lnc-OPHN1-5 expression decreased the AR protein expression (Fig. 3B). In addition, by applying the MMTV-luciferase promoter-reporter assay, we found that knocking down lnc-OPHN1-5 expression enhanced AR transactivation and increasing lnc-OPHN1-5 expression led to suppress AR transactivation (Fig. 3F, G). On a molecular level, the RT-PCR assay suggested that lnc-OPHN1-5 did not influence AR mRNA level (Fig. 3H, I), but the downstream genes, such as *PSA, FKBP5*, and *TMPRSS2*, were significantly increased after silencing the expression of this lncRNA (Fig. 3J).

Taken together, the results from Fig. 3 suggest that lnc-OPHN1-5 may alter the AR transactivation as well as AR protein expression, without influencing the AR protein stability and AR mRNA expression.

### Mechanism dissection of how lnc-OPHN1-5 can decrease AR protein expression: via reducing AR translation

**Fig. 4:**
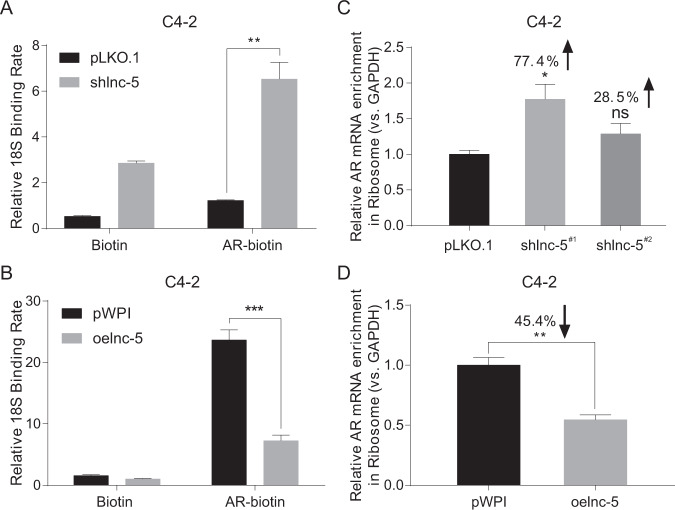
The lnc-OPHN1-5 (lnc-5) expression influences the AR mRNA translation. **A**, **B** Knocking down lnc-5 expression (shlnc-5) increased the interaction between AR mRNA and 18S (A), while their interaction could be suppressed by ectopic lnc-5 (oelnc-5) expression in C4-2 cells by RNA immunoprecipitation (RIP) and reverse transcription-polymerase chain reaction (RT-PCR) assays (**B**). **C** Shlnc-5 expression increased the enrichment of AR mRNA in ribosome in C4-2 cells determined by RT-PCR assay. **D** Oelnc-5 expression decreased the enrichment of AR mRNA in ribosome in C4-2 cells determined by RT-PCR assay. *P < 0.05; **P < 0.01; ***P < 0.001; ns, no significant difference.

As shlnc-OPHN1-5 increased AR protein expression, yet failed to alter AR protein stability or mRNA expression, we tested whether AR mRNA translation was enhanced. To determine the association of AR mRNA with ribosomes, thus translation under the influence of lnc-OPHN1-5, we extracted the AR mRNA through biotin-labeled antisense AR oligonucleotide (Oligo), and examined the associated ribosome through the measurement of 18S ribosomal RNA, and results revealed that knocking down lnc-OPHN1-5 expression led to significantly increase the abundance of ribosomes that were associated with AR mRNA, and increasing lnc-OPHN1-5 expression led to suppress their interaction (Fig. 4A, B).

Furthermore, in the ribosome fraction upon cellular organelle fractionation, we found that knocking down lnc-OPHN1-5 expression led to increasing the abundance of AR mRNA, while overexpressing lnc-OPHN1-5 expression decreased AR mRNA in the ribosome fraction (Fig. 4C, D respectively), consistent with the AR mRNA pull-down results. Taken together, the results from Fig. 4, suggest that lnc-OPHN1-5 decreases AR protein expression potentially via reducing AR protein translation.

### Mechanism dissection of how lnc-OPHN1-5 reduces AR mRNA translation: via suppressing AR mRNA-hnRNPA1 interaction

**Fig. 5:**
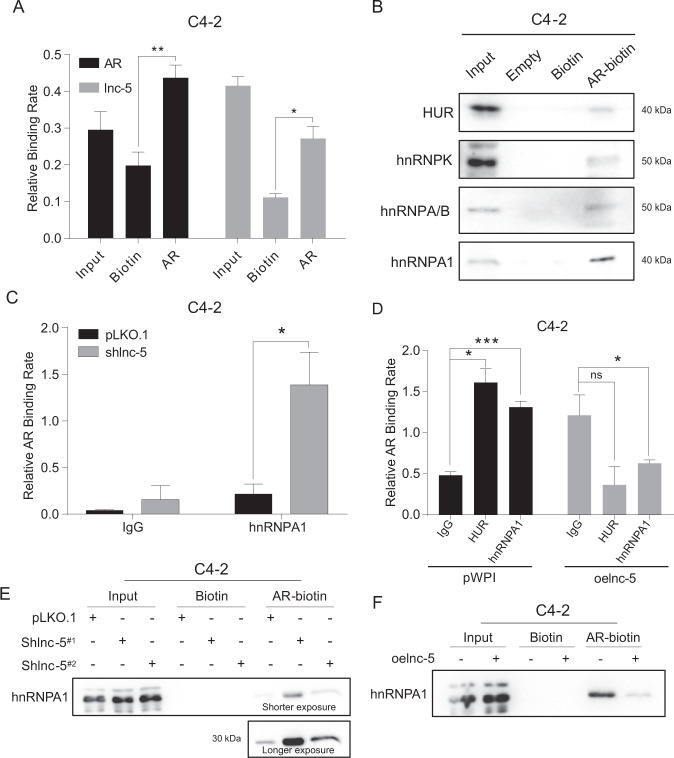
The lnc-OPHN1-5 (lnc-5) influences the AR mRNA interaction with RNA-binding protein (RBP) hnRNPA1 and ribosome RNA 18S. **A** RNA immunoprecipitation (RIP) assay by pull-down of AR mRNA using its specific biotin showed that AR directly interacted with lnc-5 in C4-2 cells determined by reverse transcription-polymerase chain reaction (RT-PCR). **B** RIP and western blotting (WB) assays showed that AR mRNA directly interacted with four RBPs, including hnRNPA1, HUR, hnRNPK, and hnRNPA/B. **C**, **D** RIP assay by pull-down of hnRNPA1 showed that knocking down lnc-5 (shlnc-5) increased the interaction between AR mRNA and hnRNPA1 (**C**) while their interaction could be suppressed (**D**) by ectopic lnc-5 (oelnc-5) expression in C4-2 cells determined by RT-PCR assay. **E**, **F** RIP assay by pull-down of AR mRNA biotin showed that shlnc-5 (with shlnc-5^#1^ and shlnc-5^#2^) increased the interaction between AR mRNA and hnRNPA1 while their interaction could be suppressed by oelnc-5 expression in C4-2 cells determined by RIP and WB assays. *P < 0.05; **P < 0.01, ***P < 0.001; ns, no significant difference.

Recent studies indicated that mRNA translation could be influenced by mRNA’s interaction with lncRNAs as well as RBPs [14, 35]. Based on the published literature, we selected four common RBPs that have been found to be associated with PCa, including hnRNPA1 [36], hnRNPA/B [37], hnRNPK [38], and HUR [39]. Indeed, we used biotin-labeled antisense oligos to pull-down the AR mRNA (AR-biotin) and its interacting molecules, including RNAs or proteins. As indicated in Fig. 5A, we found that AR mRNA was successfully pulled-down by AR-biotin, and we detected the presence of lnc-OPHN1-5, as well as the four RBPs mentioned above (Fig. 5B). As the previous study suggested that hnRNPA1 influences Enz treatment sensitivity [40], we chose hnRNPA1 as a representative for further investigation.

The RIP assay by pull-down of hnRNPA1 using its specific antibody was applied to detect whether knocking down (Fig. S4A) or increasing (Fig. S4B) lnc-OPHN1-5 expression influenced the interaction between hnRNPA1 and AR mRNA. These results suggested that knocking down lnc-OPHN1-5 increased the interaction between AR mRNA and hnRNPA1 (Fig. 5C), while their interaction was decreased after increasing lnc-OPHN1-5 expression (Fig. 5D). Similar results were also obtained while replacing the hnRNPA1 antibody with biotin-labeled antisense of AR mRNA (Fig. 5E, F).

Taken together, the data from Fig. 5 and Fig. S4A, B suggest that lnc-OPHN1-5 suppresses hnRNPA1-AR mRNA interaction, thus decreasing AR mRNA translation to decrease AR protein. Such a consequence may then lead to an increase in Enz treatment sensitivity.

### The lnc-OPHN1-5 competes with hnRNPA1 binding to 3′UTR of AR mRNA to decrease its translation

**Fig. 6:**
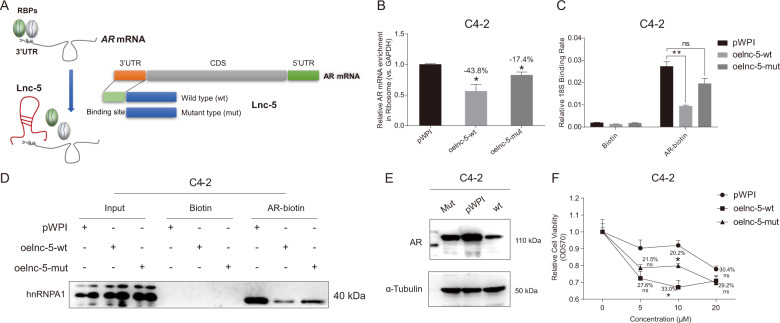
The lnc-OPHN1-5 (lnc-5) binding to the specific region of AR mRNA to influence its translation, protein expression, and Enzalutamide (Enz) treatment sensitivity. **A** The cartoon shows the construction of a mutant (mut)-lnc-5 by deleting the AR mRNA-binding site. **B** The influence of ectopic wild type wt/mut-lnc-5 (oelnc-5-wt, oelnc-5-mut) expression on the enrichment of AR mRNA in ribosomes in C4-2 cells determined by reverse transcription-polymerase chain reaction (RT-PCR) assay. **C** The influence of oelnc-5-wt/mut expression on the interaction between AR mRNA and 18S in C4-2 cells determined by RT-PCR assay. **D** The influence of oelnc-5-wt/mut expression on the interaction between AR mRNA and hnRNPA1 in C4-2 cells determined by western blotting (WB) assay. **E** The influence of oelnc-5-wt/mut expression on AR protein levels in C4-2 cells determined by WB assay. **F** The influence of oelnc-5-wt/mut expression on Enz treatment sensitivity in C4-2 cells determined by MTT assay. *P < 0.05; **P < 0.01; ***P < 0.001; ns, no significant difference; RBP, RNA binding protein; UTR, untranslated regions.

We found that lnc-OPHN1-5 directly interacted with the 3’UTR of the AR mRNA (Table S4), according to the website prediction (https://blast.ncbi.nlm.nih.gov/Blast.cgi?PROGRAM = blastn&PAGE_TYPE = BlastSearch&LINK_LOC = blasthome). To test whether this binding was critical for the lnc-OPHN1-5 function, we then constructed a mutant (mut) lnc-OPHN1-5 by deleting the presumptive AR-mRNA interaction regions (Fig. 6A). After ectopic expression of wild-type (wt) and mut-lnc-OPHN1-5, we found the mut-lnc-OPHN1-5 failed to significantly decrease the enrichment of AR mRNA in the ribosome, as well as the interaction between AR mRNA and 18S ribosomal RNA compared with the wt-lnc-OPHN1-5 (Fig. 6B, C). In addition, RIP and western blotting assays revealed that only wt-lnc-OPHN1-5 effectively suppressed AR mRNA-hnRNPA1 interaction (Fig. 6D) and AR protein expression (Fig. 6E and Fig. S4C), thus significantly increased Enz treatment sensitivity, while the mut lncRNA had less capacity to do so (Fig. 6F).

Taken together, the data from Fig. 6, Table S4, and Fig. S4C, suggest that lnc-OPHN1-5 competes with hnRNPA1 binding to 3’UTR of AR mRNA to decrease its translation and protein level.

### Preclinical studies with multiple PCa in vitro cell lines and in vivo mouse model demonstrate that targeting the lnc-OPHN1-5/AR/hnRNPA1 complex with shhnRNPA1 leads to increase Enz treatment sensitivity to better suppress PCa progression

**Fig. 7:**
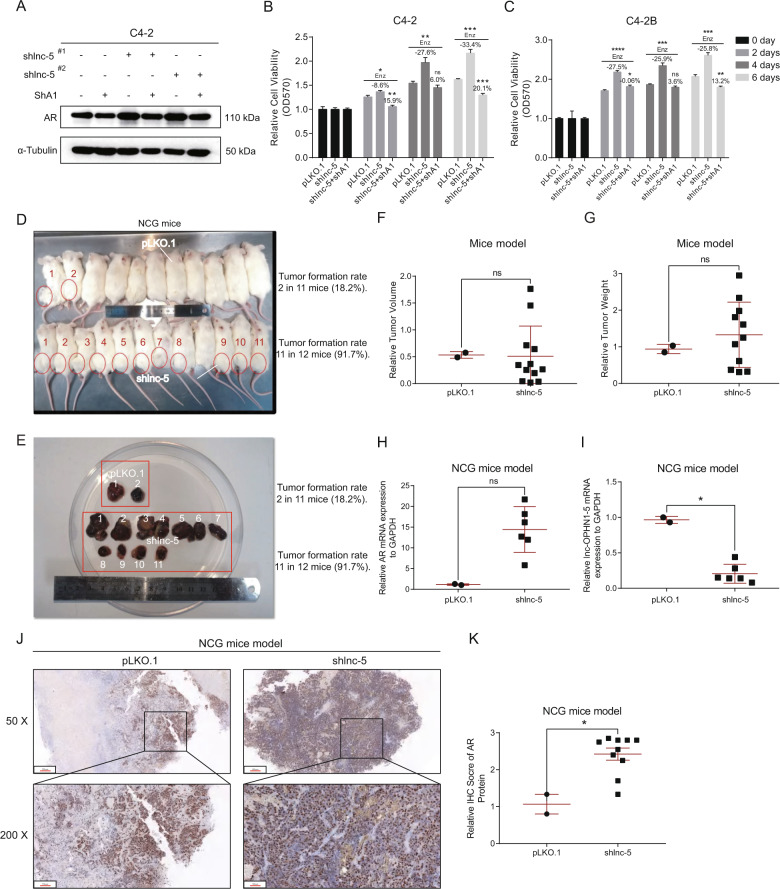
Targeting the lnc-OPHN1-5 (lnc-5)/AR/hnRNPA1 complex with shhnRNPA1 led to increasing Enzalutamide (Enz)-treatment sensitivity and in vivo evidence of lnc-5’s function in prostate cancer (PCa). **A** Knocking down hnRNPA1 (shhnRNPA1) expression reversed silencing lnc-5 (shlnc-5^#1^ and shlnc-5^#2^) expression induced AR up-regulation in C4-2 cells determined by western blotting (WB) assay. B-C. ShhnRNPA1 expression re-sensitized shlnc-5 caused PCa Enz-resistance in C4-2 (**B**) and C4-2B cells determined by MTT assay (**C**). **D**, **E** Display of the mice images (**D**) and formed tumors (**E**) in vector and shlnc-5 subgroups. **F**, **G** The comparison of tumor volumes (**F**) and tumor weights (**G**) between the shlnc-5 and vector subgroups. **H**, **I** Reverse transcription-polymerase chain reaction (RT-PCR) results showing the expression of AR (**H**) and lnc-5 (**I**) between the shlnc-5 and vector subgroups. **J**, **K** Immunohistochemistry (IHC) assay showing the AR protein expression between the shlnc-5 and vector subgroups (**J**), and the immunohistochemistry (IHC) scores were also quantified (**K**). *P < 0.05; **P < 0.01; ***P < 0.001; ns, no significant difference; NCG mice, NOD CRISPR Prkdc Il2r Gamma triple-immunodeficient mice.

To link our above studies to potential clinical applications, we were interested in applying small molecule(s) to target this newly identified lnc-OPHN1-5/AR/hnRNPA1 complex to increase the Enz treatment sensitivity, thus better suppress the PCa progression. We first applied the shhnRNPA1 to reverse/block the lnc-OPHN1-5 increased AR protein expression (Fig. 7A). The results revealed that adding shhnRNPA1 increased Enz treatment sensitivity in C4-2 and C4-2B cells (Fig. 7B, C).

We further examined whether the lnc-OPHN1-5 could influence the Enz treatment sensitivity or tumor growth in the in vivo mice model. C4-2 cells were lentivirally infected with pLKO.1 and shlnc-OPHN1-5 constructs. These cells (3 × 106) were mixed with Matrigel (1:1, v/v) and subcutaneously injected into the left hind limb of the NCG male mice at six weeks of age. Two months later, we found that only two if 12 mice formed tumors in the pLKO.1 group (2/11, 18.2%), while 11 of 12 mice developed tumors in the shlnc-OPHN1-5 group (11/12, 91.7%, *χ*^2^ = 9.798, *P* = 0.0017) (Fig. 7D, mice images, and E, tumor formation rates). Further analyses found no significant difference in tumor volumes (Fig. 7F) and tumor weights (Fig. 7G) between the two groups, using Mann–Whitney tests, *P* > 0.05. The RT-PCR assay found that the expression of lnc-OPHN1-5 was significantly lower in the tumors of the shlnc-OPHN1-5 group compared with vector controls (Mann–Whitney tests, *P* < 0.05, Fig. 7I). The AR mRNA expression was conversely higher (Fig. 7H), but lack statistical significance according to Mann–Whitney tests, *P* = 0.0714. In addition, IHC analysis showed that AR protein was higher in tumors with the expression of the shlnc-OPHN1-5 group (Fig. 7J) compared with vector control (Fig. 7K), using Mann–Whitney tests, *P* < 0.05.

**Fig. 8:**
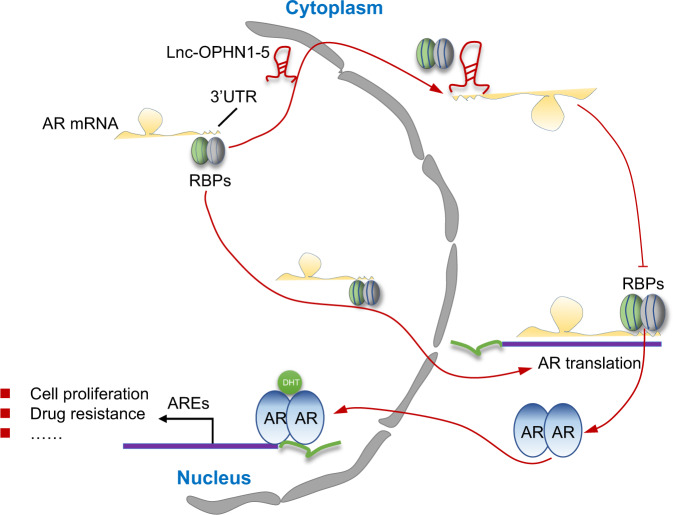
Schematic model. The lnc-OPHN1-5 (lnc-5) directly interacts with AR mRNA, reducing AR mRNA interaction with RNA binding proteins (RBPs), thus decreasing AR mRNA translation, protein expression, and transcriptional activity, enhancing Enzalutamide (Enz) treatment sensitivity. ARE, androgen receptor response element; UTR, untranslated region.

Taken together, the data from Figs. 1–7, Tables S1–S4, and Figs. S1–4 suggest that lnc-OPHN1-5 interacts with the specific region of AR mRNA 3’UTR, reducing the latter’s interaction with RBP-hnRNPA1, thus decreasing AR mRNA translation and protein expression. Targeting this newly identified lnc-OPHN1-5/AR/hnRNPA1 complex with a small molecule like shhnRNPA1 could then lead to an increase in Enz sensitivity to better suppress PCa progression. A cartoon was made to illustrate the study (Fig. 8).

## Discussion

Enz has been in clinical use to extend mCRPC patients’ survival an extra 4.8 months [2–4]. However, most patients still succumb after the development of Enz-resistance. Currently, several mechanisms have been proven to be involved in the Enz-resistance in CRPC, including induction of altered glucocorticoid receptor [41], AKT signals [42–44], AR-v7 expression [45], and AR (F876L) mutations [46–48]. In addition, enhanced AR protein levels or signals also have been proven to lead to Enz-resistance [7, 34].

Han et al. [34] found that Triptolide suppresses AR binding to the promoter regions of the AR target genes. In addition, the low dose of Triptolide enhances the anti-androgen Enz efficacy on CRPC xenografts with minimal side effects. In Takeda et al*.’*s work [7], they characterize a somatically acquired AR enhancer located 650 kb centromeric to the AR. Systematic perturbation of this enhancer decreases AR protein expression, decreasing cell viability through a genome editing method, while inserting an additional copy of this region increases PCa cell proliferation under low-androgen conditions and compromises Enz efficacy. Both of these studies suggested that enhanced AR level and signal activity are among the mechanisms for Enz treatment resistance.

Thus, we hypothesized that there might exist some lncRNAs that are located surrounding the *AR* gene on chromosome X, and these lncRNAs potentially have a role on AR function. Notably, we found that only lnc-OPHN1-5 suppresses AR expression and associates with enhanced Enz treatment sensitivity. In addition, evidence from resistant cell lines, the PDX mouse models, PCa tissues, and a human PCa study all indicated that PCa patients with low expression of lnc-OPHN1-5 likely have unfavorable prognoses and probably are less sensitive to Enz treatment. Consistently, our in vivo data shows a vast difference in tumor formation rate between the vector group and shlnc-OPHN1-5 group (18.2% vs. 91.7%, *χ*^2^ = 9.798, *P* = 0.0017), further supporting the notion that the expression of lnc-OPHN1-5 is negatively associated with PCa patients’ poor prognosis. The mechanism study found that the lnc-OPHN1-5 directly interacts with the AR mRNA, reducing the interaction between AR mRNA and hnRNPA1, therefore, suppresses the AR mRNA translation and protein synthesis, enhancing the Enz treatment sensitivity. Consistent with that, knocking down hnRNPA1 reverses shlnc-OPHN1-5 induced AR protein expression and re-sensitizes cells to Enz treatment.

The involvement of hnRNPA1 in mRNA splicing has been recognized for a long time [49, 50], but its role in regulating the translational process has only recently been appreciated [51–53]. During tumor development, several oncogenes and tumor promoters are translationally controlled by hnRNPA1, including FGF-2, c-MYC, CCND1, XIAP, BCL-XL [52, 54, 55], *etc*. Nadiminty et al. [40] found that silencing hnRNPA1 and consequently of AR-v7 re-sensitizes cells to Enz treatment, indicating that upregulation of hnRNPA1 may confer resistance to anti-androgen therapies by promoting expression of the AR variants. However, we found that silencing lnc-OPHN1-5 expression did not influence the AR-v7 expression. Notably, we found that hnRNPA1 is involved in the lnc-OPHN1-5 mediated AR mRNA translation process. Ectopic lncRNA expression significantly suppresses the interaction between AR mRNA and hnRNPA1, thus decreases AR protein expression. The opposite phenomenon was observed when we knocked down lnc-OPHN1-5 expression. These conclusions were supported by the AR mRNA abundance in the ribosomal fraction as well as ribosomal RNA level in the AR mRNA pull-down assay using biotin-conjugated antisense oligos. Furthermore, our results also found that knocking down hnRNPA1 decreases shlnc-OPHN1-5 induced AR protein increase and re-sensitizes C4-2 cells to Enz treatment. Altogether with the previous report, our data suggested that lnc-OPHN1-5 is a part of the complex regulation of AR protein expression involving the hnRNPA1-mediated mRNA translation.

One weakness of our work is that we did not provide evidence to demonstrate the Enz sensitivity in the mice xenograft model due to the inefficiency, therefore no statistical power of tumor formation with the parental cells. Similarly, clinical samples from the existing GEO dataset are limited to provide statistical power to support our hypothesis that low expression of lnc-OPHN1-5 links to unfavorable Enz treatment response. More clinical samples before and after Enz treatment are needed to test this hypothesis in PCa patients rigorously. In addition, since Enz treatment sensitivity might be influenced by cell growth rates at a certain timepoint, in our case, the cell growth could be slightly decreased by the lnc-OPHN1-5 expression. The caveat notwithstanding we have the evidence to support the role of lnc-OPHN1-5 from clinical samples, in vivo mice models, and PDX xenograft models, we still need more evidence to rigorously demonstrate that its impact on Enz treatment sensitivity of PCa is not derived from its minor impact on cell proliferation.

In summary, we characterized a novel lncRNA, lnc-OPHN1-5, which influences anti-androgen Enz treatment sensitivity by decreasing AR mRNA translation and protein synthesis. Targeting the lnc-OPHN1-5/hnRNPA1/AR axis represents a potential strategy for the treatment of CRPC.

## Supplementary information

Legends of Supplementary Figures and Tables

Fig. S1

Fig. S2

Fig. S3

Fig. S4

Table S1

Table S2

Table S3

Table S4

## Data Availability

The data used and/or analyzed during the current study are available from the corresponding author on reasonable request.
