# Supplementary material for: Targeting the Lnc-OPHN1-5/androgen receptor/hnRNPA1 complex increases Enzalutamide sensitivity to better suppress prostate cancer progression
Source: Cell Death Dis. 2021 Sep 20;12(10):855. doi: 10.1038/s41419-021-03966-4 (PMC8452728; doi:10.1038/s41419-021-03966-4)
Supplement: Supplementary file 1 — Legends of Supplementary Figures and Tables [file 41419_2021_3966_MOESM1_ESM.docx]

**Supplementary Information (Supplementary Figure and Table Legends)**

**Fig. S1. The aberration landscape of AR in prostate cancer (PCa) patients. A.** The aberrant status of AR in different PCa cohorts. **B.** Kaplan-Meier plot and log-rank analysis suggested that AR aberrations were associated with worse overall survival outcomes. **C.** Kaplan-Meier plot and log-rank analysis suggested that AR aberrations were associated with worse recurrence-free survival outcomes. **D.** The gene mutation difference between AR altered and unaltered groups. **E.** The specific location domain of these point mutations on AR. **Note:** Prostate (SU2C) (PMID: 30537516); Prostate (SU2C 2019) (PMID: 31061129); Prostate (FHCRC, 2016) (PMID: 26928463); Prostate (MICH) (PMID: 22722839); NEPC (Multi-Institute 2016) (PMID: 26855148); MSK-IMPACT Prostate (PMID: 32317181); PRAD (MSKCC/DFCI 2018) (PMID: 29610475); The MPC Project (unpublished, https://www.cbioportal.org/study/summary?id=prad_mpcproject_2018); Prostate (MSKCC 2010) (PMID: 20579941); PRAD_MCSPC (MSK, CCR 2020) (PMID: 32220891); Prostate (TCGA PanCan 2018) (PMID: 29625048, 29596782, 29622463, 29617662, 29625055, 29625050, 29617662, 30643250, 32214244, 29625049, 29850653); Prostate (MSKCC 2014) (PMID: 25201530); Prostate Organoids (PMID: 25201530); PROSTATE (MSK 2019) (PMID: 31564440); Prostate (DKFZ) (PMID: 30537516). AR-alteration is defined as changes incurred in the AR gene (missense, inframe insertion, inframe deletion, nonsense, frameshift insertion, frameshift deletion, nonstart, nonstop, splicing, others), structural variants/fusions, or copy number alterations (amplification and deletion), while AR-unaltered means that the AR gene did not have the occurrence of any mutation, structural variant/fusion, or copy number alterations.

**Fig. S2. The lnc-OPHN1-5 (lnc-5) influences prostate cancer (PCa) cell Enzalutamide (Enz) treatment sensitivity.** C4-2 (**A**) and C4-2R (**B**) cells transfected with pLKO.1 and shlnc-5 lentivirus, and then the cell proliferation was determined by Ethynyl-2-deoxyuridine (EdU) staining assays. **C-D.** Matched quantification data of EdU staining for C4-2 (**C**) and C4-2R (**D**) cells. *, *P* < 0.05, ns, no significant difference.

**Fig. S3. The functional role of lnc-OPHN1-5 (lnc-5) and its subcellular location. A.** The knock down efficiency of lnc-5^#1^ (shlnc-5^#1^) in C4-2 cells determined by reverse transcription-polymerase chain reaction (RT-PCR) assay. **B.** Knocking down lnc-5^#1^ expression decreased Enzalutamide (Enz) treatment sensitivity in C4-2 cells, determined by MTT assay, a result consistent with the shlnc- 5^#2^ listed in **Fig. 1**. **C.** The protein-coding potential prediction of lnc-5 extracted from LNCipedia database (version 5.2: <https://lncipedia.org/db/transcript/lnc-OPHN1-5:1>). **D.** Since the function of lncRNAs is related to their sublocation, we used nuclear-plasma separation and RT-PCR assays to verify the sublocation of lnc-5 in PCa cells. The MALAT1 and α-Tubulin were used to reflect nuclear and cytoplasm (or cytosol) purity, respectively. **E.** The relative expression (Fragments Per Kilobase per Million, FPKM) of lnc-OPHN1-5 in PCa patients pre- androgen deprivation therapy (ADT) vs. post-ADT extracted from Gene Expression Omnibus (GEO) dataset (GSE48403). *, *P* < 0.05; ***, *P* < 0.001; ns, no significant difference.

**Fig. S4. Pull-down efficiencies. A-B.** Pull-down hnRNPA1 efficiencies after (**A**) knocking down lnc-OPHN1-5 (shlnc-5) or (**B**) ectopic lnc-OPHN1-5 (oelnc-5) expression determined by RIP and western blotting (WB) assays. **C.** Overexpression efficiencies of wild-type (wt) and mutant (mut) lnc-5 in C4-2 cells determined by RT-PCR assays. ***, *P* < 0.001.

**Table S1. Plasmid information used in current work.**

**Table S2. The correlation between lnc-OPHN1-5 (lnc-5) expression and clinicopathological features in prostate cancer (PCa) patients.**

**Table S3. Prostate cancer (PCa) patient demographics of GSE22260 cohort.**

**Table S4. Detailed information of the lnc-OPHN1-5 (lnc-5) and the interaction sites with AR mRNA.**
