# Supplementary figures and images for: Targeting the Lnc-OPHN1-5/androgen receptor/hnRNPA1 complex increases Enzalutamide sensitivity to better suppress prostate cancer progression
Source: Cell Death Dis. 2021 Sep 20;12(10):855. doi: 10.1038/s41419-021-03966-4 (PMC8452728; doi:10.1038/s41419-021-03966-4)

Fig. S1

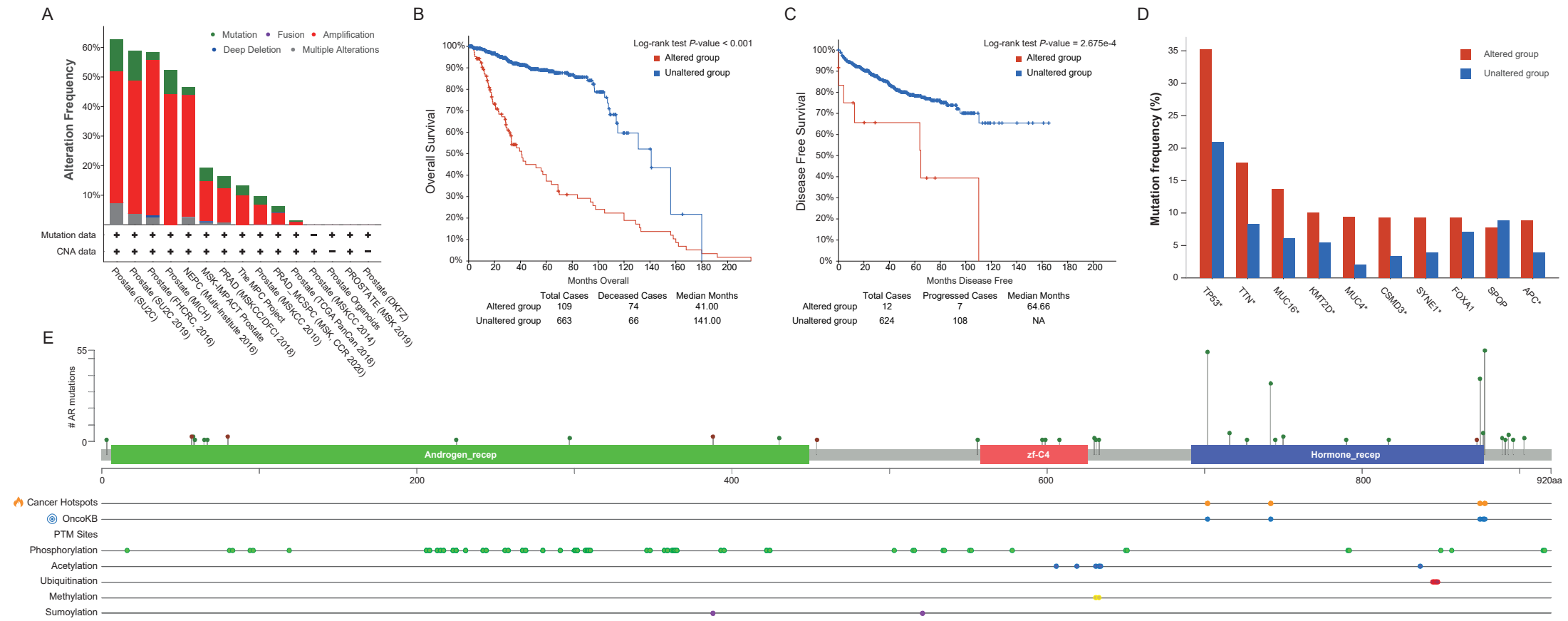

Supplement: Supplementary file 2 — Fig. S1 [file 41419_2021_3966_MOESM2_ESM.pdf]

# Fig. S2

A

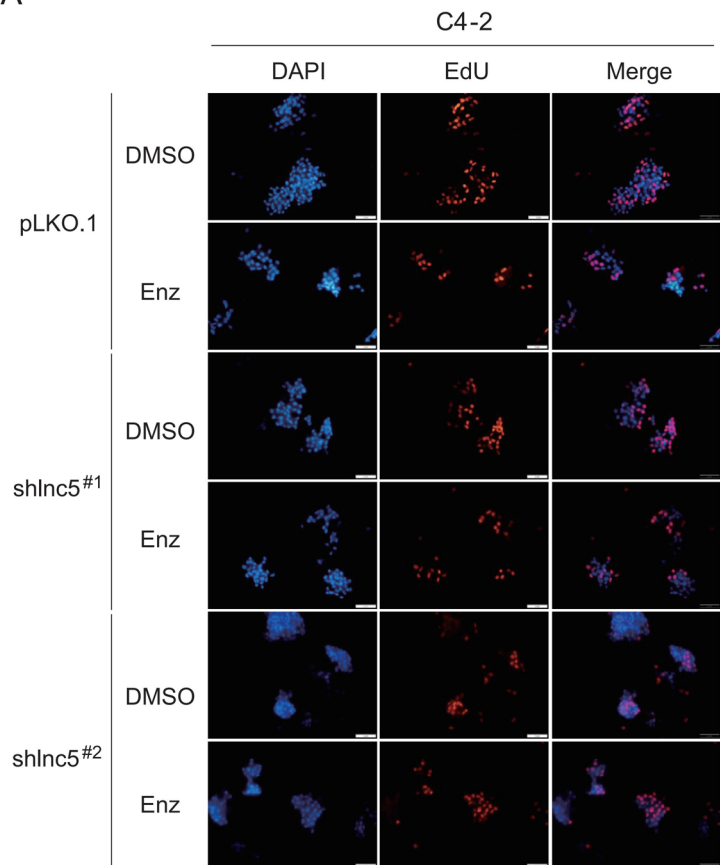

B

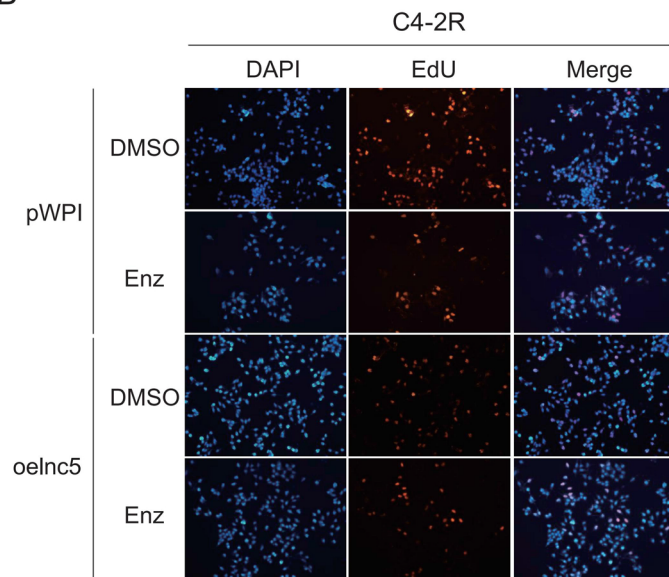

D

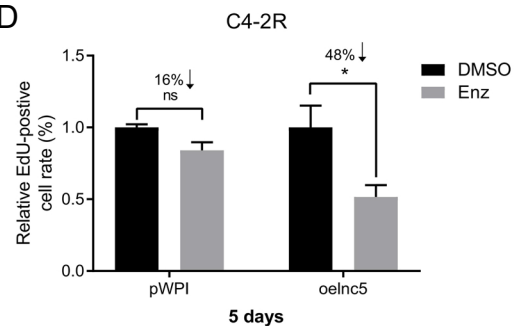

C

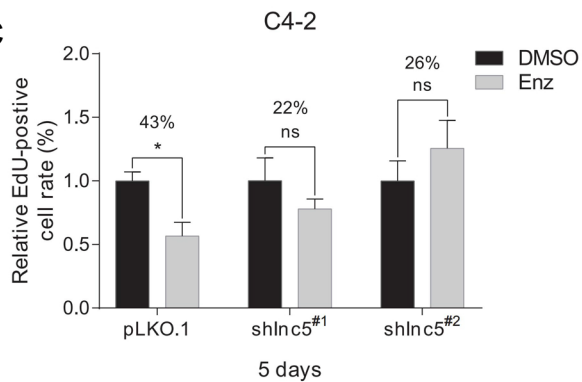

Supplement: Supplementary file 3 — Fig. S2 [file 41419_2021_3966_MOESM3_ESM.pdf]

Fig. S4

A

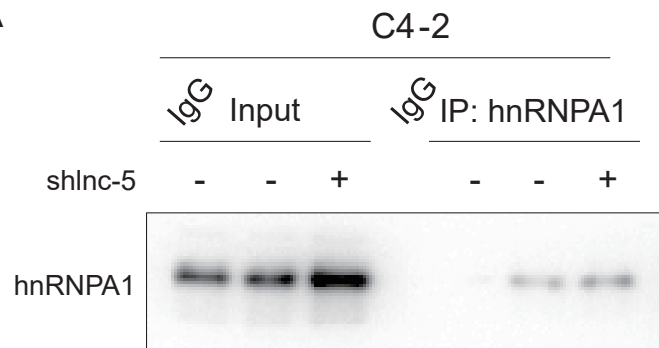

B

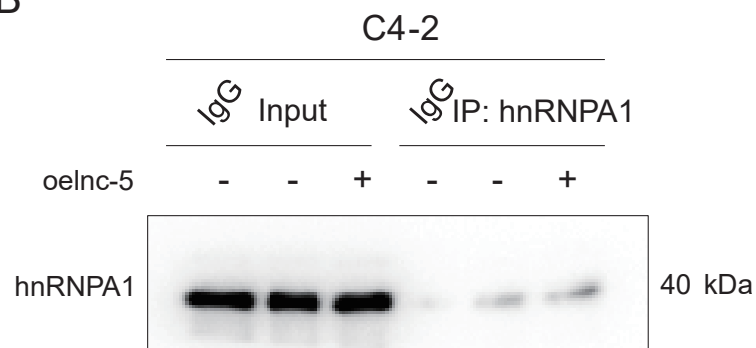

C

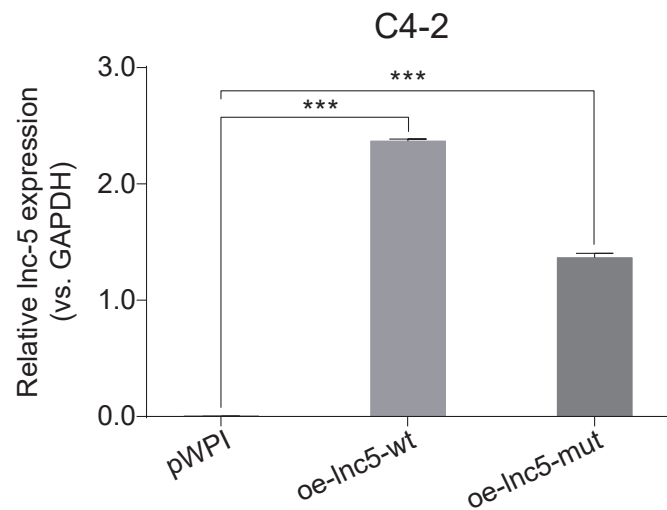

Supplement: Supplementary file 5 — Fig. S4 [file 41419_2021_3966_MOESM5_ESM.pdf]
