# Supplementary material for: Targeting the Lnc-OPHN1-5/androgen receptor/hnRNPA1 complex increases Enzalutamide sensitivity to better suppress prostate cancer progression
Source: Cell Death Dis. 2021 Sep 20;12(10):855. doi: 10.1038/s41419-021-03966-4 (PMC8452728; doi:10.1038/s41419-021-03966-4)

Fig. S3

A

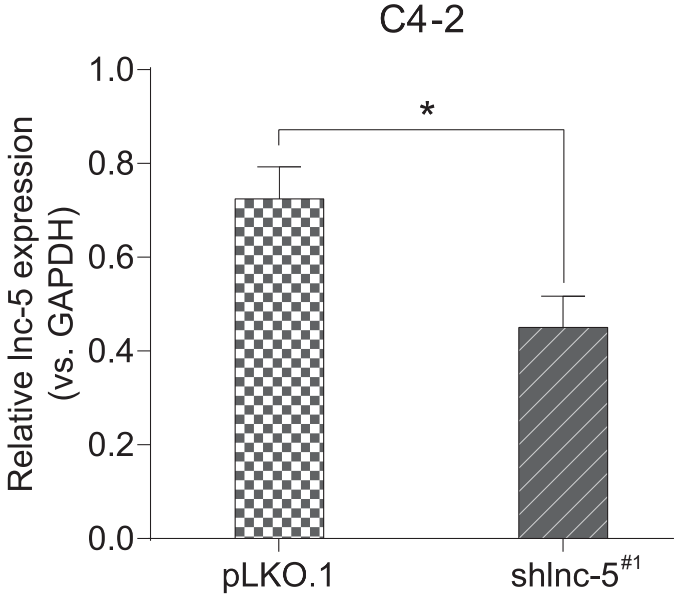

B

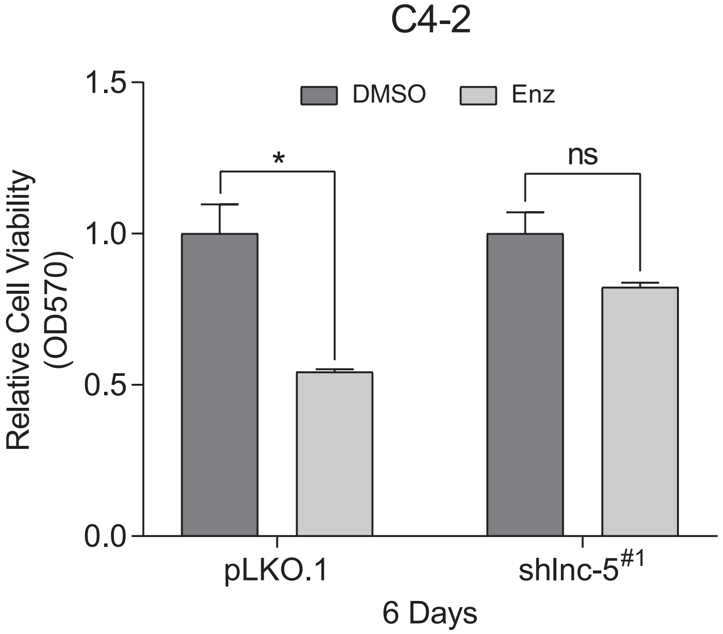

C

Protein coding potential

Metric	Raw results	Interpretation
PRIDE reprocessing 2.0	0	non-coding
Lee translation initiation sites	0	non-coding
PhyloCSF score	-112.9597	non-coding
CPAT coding probability	3.45%	non-coding
Bazzini small ORFs	0	non-coding

D

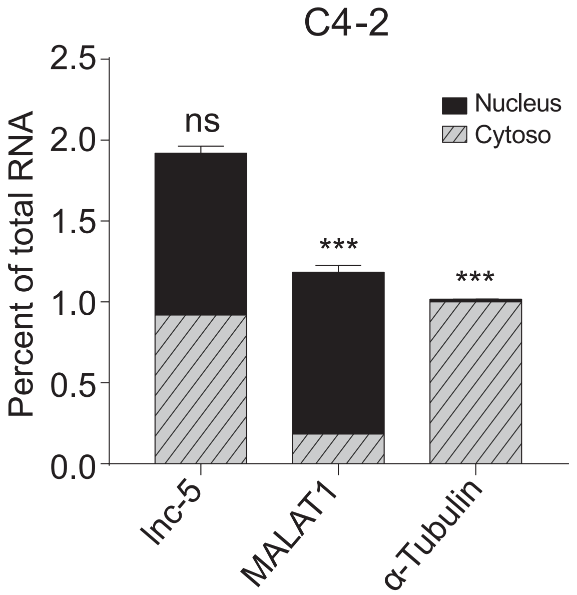

E

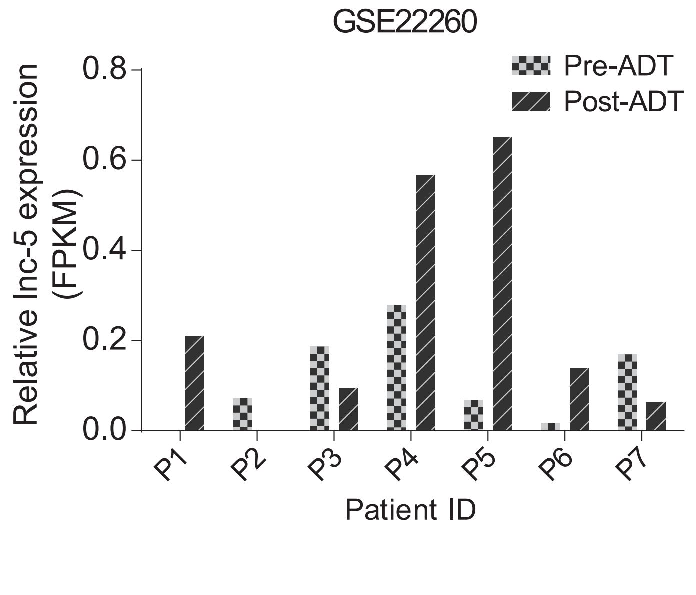

Supplement: Supplementary file 4 — Fig. S3 [file 41419_2021_3966_MOESM4_ESM.pdf]
