# Supplementary material for: Targeting the Lnc-OPHN1-5/androgen receptor/hnRNPA1 complex increases Enzalutamide sensitivity to better suppress prostate cancer progression
Source: Cell Death Dis. 2021 Sep 20;12(10):855. doi: 10.1038/s41419-021-03966-4 (PMC8452728; doi:10.1038/s41419-021-03966-4)
Supplement: Supplementary file 6 — Table S1 [file 41419_2021_3966_MOESM6_ESM.docx]

**Table S1. Plasmid information used in current work.**

| **Gene_ID** | **Vector** | **Application** | **Sequence** |
| --- | --- | --- | --- |
| Lnc-AR-1 | pLKO.1 | Knocking down | **F:** CCGGUUUAUAUUCAUCUGCUUGCGGATCCGCAAGCAGATGAATATAAATTTTTG |
|  |  |  | **R:** AATTCAAAAAUUUAUAUUCAUCUGCUUGCGGATCCGCAAGCAGATGAATATAAA |
| Lnc-AR-2 | pLKO.1 | Knocking down | **F:** CCGGUUUAACAAGGAUAUCCAGCGGATCCGCTGGATATCCTTGTTAAATTTTTG |
|  |  |  | **R:** AATTCAAAAAUUUAACAAGGAUAUCCAGCGGATCCGCTGGATATCCTTGTTAAA |
| Lnc-OPHN1-1 | pLKO.1 | Knocking down | **F:** CCGGUUUAUAUUUGAAUAUAUGCGGATCCGCATATATTCAAATATAAATTTTTG |
|  |  |  | **R:** AATTCAAAAAUUUAUAUUUGAAUAUAUGCGGATCCGCATATATTCAAATATAAA |
| Lnc-OPHN1-5^#1^ | pLKO.1 | Knocking down | **F:** CCGGUAUAUUUAUCCUUUAAAGCGGATCCGCTTTAAAGGATAAATATATTTTTG |
|  |  |  | **R:** AATTCAAAAAUAUAUUUAUCCUUUAAAGCGGATCCGCTTTAAAGGATAAATATA |
| Lnc-OPHN1-5^#2^ | pLKO.1 | Knocking down | **F:** CCGGUUAUAAUUCUCUUAUCUUCGGATCCGAAGATAAGAGAATTATAATTTTTG |
|  |  |  | **R:** AATTCAAAAAUUAUAAUUCUCUUAUCUUCGGATCCGAAGATAAGAGAATTATAA |
|  | pWPI | Overexpression | **F:** TTTCGACATTTAAATTTAATATGCATTGAGCAATCGGAGTC |
|  |  |  | **R:** ATTCCTGCAGCCCGTAGTTTACCTAAGAATGAAGTTTCTAA |
| HnRNPA1 | pLKO.1 | Knocking down | **F:** CCGGGTCTGATCGTGACGCTGAATAGGATCCTATTCAGCGTCACGATCAGACTTTTTG |
|  |  |  | **R:** AATTCAAAAAGTCTGATCGTGACGCTGAATAGGATCCTATTCAGCGTCACGATCAGAC |
| HnRNPK | pLKO.1 | Knocking down | **F:** CCGGTGATCTTGGTGGACCTATTATGGATCCATAATAGGTCCACCAAGATCATTTTTG |
|  |  |  | **R:** AATTCAAAAATGATCTTGGTGGACCTATTATGGATCCATAATAGGTCCACCAAGATCA |
| HUR | pLKO.1 | Knocking down | **F:** CCGGGAGAACGAATTTGATCGTCAAGGATCCTTGACGATCAAATTCGTTCTCTTTTTG |
|  |  |  | **R:** AATTCAAAAAGAGAACGAATTTGATCGTCAAGGATCCTTGACGATCAAATTCGTTCTC |
