# Supplementary material for: Targeting the Lnc-OPHN1-5/androgen receptor/hnRNPA1 complex increases Enzalutamide sensitivity to better suppress prostate cancer progression
Source: Cell Death Dis. 2021 Sep 20;12(10):855. doi: 10.1038/s41419-021-03966-4 (PMC8452728; doi:10.1038/s41419-021-03966-4)
Supplement: Supplementary file 7 — Table S2 [file 41419_2021_3966_MOESM7_ESM.docx]

**Table S2. The correlation between lnc-OPHN1-5 expression and clinicopathological features in prostate cancer.**

| **Parameter** | **Low-expression** | | **High-expression** | | ***P*-value** | |
| --- | --- | --- | --- | --- | --- | --- |
|  | **Number** | **%** | **Number** | **%** |  |  |
| Total | 37 | 49.3% | 38 | 50.7% |  |  |
| Age |  | |  | |  | |
| ≤71 years | 21 | 56.8% | 18 | 47.4% | 0.4159 |  |
| >71 years | 16 | 43.2% | 20 | 52.6% |  |  |
| Pathological T stage |  |  |  |  | 0.4633 |  |
| T1+T2 | 29 | 78.4% | 33 | 86.8% |  |  |
| T3+T4 | 7 | 18.9% | 5 | 13.2% |  |  |
| NA | 1 | 2.7% | 0 | 0.0% |  |  |
| Gleason score |  |  |  |  | 0.8854 |  |
| ≤7 | 22 | 59.5% | 22 | 57.9% |  |  |
| >7 | 14 | 37.8% | 15 | 39.5% |  |  |
| NA | 1 | 2.7% | 1 | 2.6% |  |  |
| BMI |  |  |  |  | 0.1335 |  |
| ≤24 | 18 | 48.6% | 25 | 65.8% |  |  |
| >24 | 19 | 51.4% | 13 | 34.2% |  |  |
| Surgical margin |  |  |  |  | 0.0930 |  |
| Positive | 8 | 21.6% | 3 | 7.9% |  |  |
| Negative | 29 | 78.4% | 35 | 92.1% |  |  |
| Recurrence |  |  |  |  | 0.9162 |  |
| No | 20 | 54.1% | 21 | 55.3% |  |  |
| Yes | 17 | 45.9% | 17 | 44.7% |  |  |
| RFS time (Mean + SEM) | 648.3 ± 66.29 | | 1022 ± 90.63 | | 0.0015 |  |

**Note:** For lymph node and distant metastases, they were rarely occurred in patients from our cohort, thus we did not focus on these issues in analyzing; NA, not available; SEM, standard error of the mean; BMI, body mass index; RFS, recurrence-free survival. We set the median values of lnc-OPHN1-5 as the cut-off to subclassify high- or low expression subgroups.
