# Supplementary material for: Targeting the Lnc-OPHN1-5/androgen receptor/hnRNPA1 complex increases Enzalutamide sensitivity to better suppress prostate cancer progression
Source: Cell Death Dis. 2021 Sep 20;12(10):855. doi: 10.1038/s41419-021-03966-4 (PMC8452728; doi:10.1038/s41419-021-03966-4)
Supplement: Supplementary file 8 — Table S3 [file 41419_2021_3966_MOESM8_ESM.docx]

**Table S3. Patient demographics of GSE22260 cohort.**

| **Patient** | **Age**  **(year)** | **KPS** | **GSS** | **TNM stage** | | | **iPSA**  **(ng/ml)** | **nPSA (ng/ml)**  **(% iPSA)** | **PFS (days)** |
| --- | --- | --- | --- | --- | --- | --- | --- | --- | --- |
|  |  |  |  | **T** | **N** | **M** |  |  |  |
| 1 | 64.7 | 100 | 8 | 3b | 0 | 0 | 370 | 3.1 (0.8) | 942 |
| 2 | 65.4 | 90 | 9 | 3b | 0 | 0 | 7.6 | 0.7 (0.1) | 155 |
| 3 | 69.6 | 100 | 8 | 3b | 1 | 0 | 5.9 | 0.7 (0.1) | 223 |
| 4 | 64.6 | 90 | 8 | 3a | 0 | 1 | 47.7 | 0.5 (1.6) | N/P |
| 5 | 51.8 | 90 | 7 | 3a | 0 | 0 | 158 | 0.4 (0.3) | N/P |
| 6 | 58.6 | 100 | 7 | 3b | 1 | 0 | 69 | 0.13 (0.2) | 489 |
| 7 | 62.9 | 90 | 7 | 3b | 1 | 0 | 32.7 | <0.02 (0.06) | N/P |

KPS = Karnofsky performance status; GSS = Gleason sum score; iPSA = initial prostate-specific antigen value at diagnosis; nPSA = nadir prostate-specific antigen value; PFS = progression-free survival; N/P = not yet progressed to date; RNA-Seq = RNA sequencing; ADT = androgen-deprivation therapy; TRUS = transrectal ultrasound.
