# Supplementary material for: Targeting the Lnc-OPHN1-5/androgen receptor/hnRNPA1 complex increases Enzalutamide sensitivity to better suppress prostate cancer progression
Source: Cell Death Dis. 2021 Sep 20;12(10):855. doi: 10.1038/s41419-021-03966-4 (PMC8452728; doi:10.1038/s41419-021-03966-4)

**Table S4. Detailed information of the lnc-OPHN1-5 and the interaction sites with AR mRNA.**

**Transcript: lnc-OPHN1-5:1**

Basic information

LNCipedia transcript_ID: lnc-OPHN1-5:1

LNCipedia gene_ID: lnc-OPHN1-5

Location (hg38): chrX: 67791955-67792895

Strand: -

Class: intergenic

Sequence Ontology term: lincRNA

Transcript size: 941bp

Exons: 1

Sources: NONCODEv4

Alternative transcript names: NONHSAT137364

**RNA sequence**

ATGCATTGAGCAATCGGAGTCAAAATCACTGAGCTAAATCCCCACTTAATGTGTGTGCTTTGTGGAGGATACTTAATTGATGCCAGAACCATAATAGAATATCTGCATTCCTTCTGTGAAACCTGTATTTCGTGTTACCTATAGATCAGCAAATATTGTCCTTTAATGTCCAAGTTCACAAAACCATTATGCTCTGAATATAACATCAGATAAAAATCCTCAATCTATTGTATACAATTACTTCCAGGCCTTTTCAGAAATAAAATGAGAAGGGATCTTTATGCATCTCTTTATTCAGCTGTTGCTGCCAATGG(query:627-640)CGCTAATGAAAATAGAGAAGTTGCAGATGAAGATAAGAGAATTATAACTGATGGCGAAACAATAAGCTTATCCATTGGATTTTTTTTTTTGACCAGAACAGAAAGTAAACAAAGACAAAGAGAAGTTTAAGGAGGAGGTGAATGATAAAAGTTATTTACAATGCTCAGCCAGAATGACTGTGATGAACCTAAGGAACATATCTTATACTTCCCAATTGATGTCATGTATGAAGAGGAATCTTTAAAGGATAAATATACATTAATGGATATTACTTACATTTATATGTGGATAAGAAATGGACTTCTTCCTTTAAAGTACGGTGTTTGACCTATTAGTAAAAGAATGAAGCT(query:277-287)TAGTTGCTGGAGAGATGAACTGACAAAAATGCTGGAGAACTGGAATGTGACTTTGG(query:221-231)AATTAACAAGGCCAATGGCCTAGTAGGAGTTATTTCCTCTAGCTCTTCTTGT(query:169-179)TTGCCTAGCTGCACTACTCCAATCCAGT(query:141-160)CTCCTCATCTTC(query:129-139)ACTTTCCTCACATGCCCAGTATTATGAATGCCACCAGGAACAGTCCCACTGGTAACTATGAACCTTTCTCTGCCAACAGACCTAAAAAATCATCAGCAAATGGGTTATTAGAAACTTCATTC(query:7-17)TTAGGT

**Delete the red one to make the mutant lnc-OPHN1-5 (Yellow marked sequences were the interaction sites).**

**Lnc-OPHN1-5 interact with AR full-length mRNA majorly on 3’UTR**


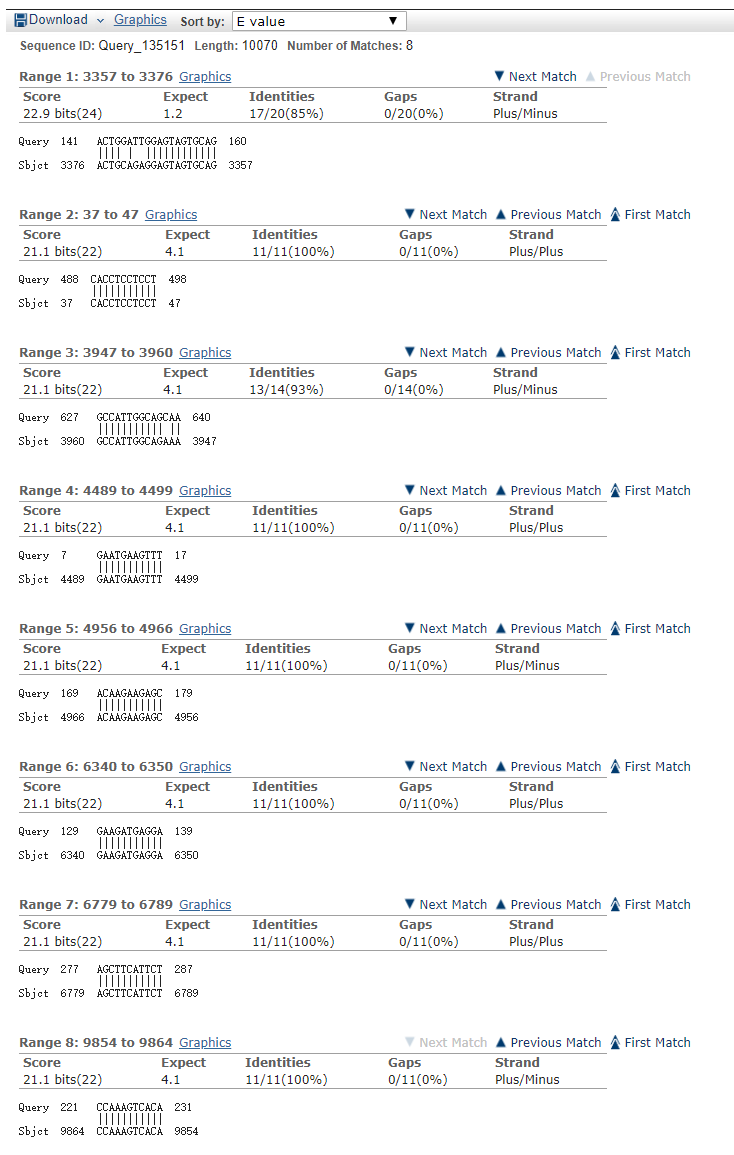


**Detail interaction sites between lnc-OPHN1-5 and AR 3’UTR**
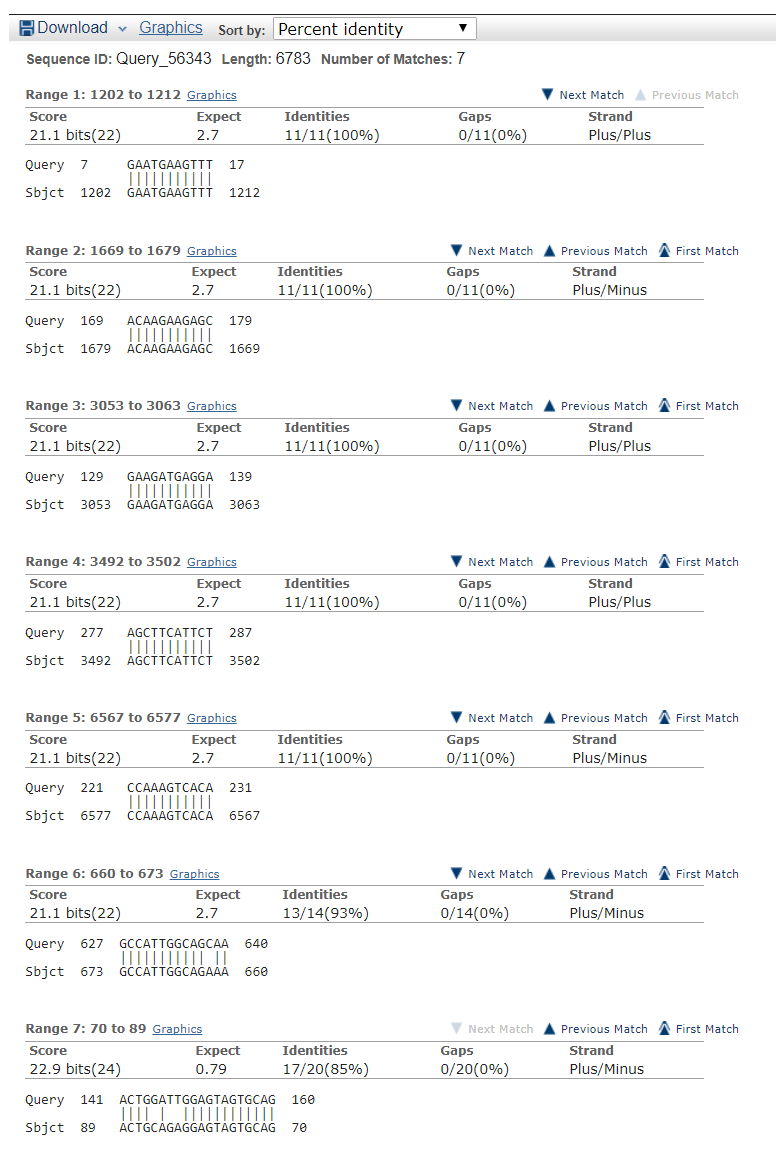

Supplement: Supplementary file 9 — Table S4 [file 41419_2021_3966_MOESM9_ESM.docx]
